# Sodium-Glucose Co-transporter-2 Inhibitor of Dapagliflozin Attenuates Myocardial Ischemia/Reperfusion Injury by Limiting NLRP3 Inflammasome Activation and Modulating Autophagy

**DOI:** 10.3389/fcvm.2021.768214

**Published:** 2022-01-10

**Authors:** Yong-Wei Yu, Jia-Qun Que, Shuai Liu, Kai-Yu Huang, Lu Qian, Ying-Bei Weng, Fang-Ning Rong, Lei Wang, Ying-Ying Zhou, Yang-Jing Xue, Kang-Ting Ji

**Affiliations:** ^1^Department of Cardiology, The Second Affiliated and Yuying Children's Hospital, Wenzhou Medical University, Wenzhou, China; ^2^Intensive Care Unit, School of Medicine, The First Affiliated Hospital, Zhejiang University, Hangzhou, China; ^3^Department of Endocrinology, The Second Affiliated and Yuying Children's Hospital, Wenzhou Medical University, Wenzhou, China

**Keywords:** sodium-glucose cotransporter-2 inhibitor, myocardial ischemia/reperfusion injury, autophagy, inflammasome, NLRP3

## Abstract

**Background:** The sodium-glucose co-transporter-2 (SGLT-2) inhibitor dapagliflozin improves cardiovascular outcomes in patients with type 2 diabetes in a manner that is partially independent of its hypoglycemic effect. These observations suggest that it may exert a cardioprotective effect by another mechanism. This study explored the effects of dapagliflozin on myocardial ischemia/reperfusion injury in a mouse model.

**Materials and Methods:** For the *in vivo* I/R studies, mice received 40 mg/kg/d dapagliflozin, starting 7 days before I/R. Evans Blue/TTC double-staining was used to determine the infarct size. Serum levels of cTnI, CK-MB, and LDH were measured. Inflammation, autophagy protein expression, and caspase-1 activity changes were measured at the protein level. Primary cardiomyocytes were used to investigate the direct effect of dapagliflozin on cardiomyocytes and to verify whether they have the same effect as observed in *in vivo* experiments.

**Result:** A high dose of dapagliflozin significantly reduced infarct size and decreased the serum levels of cTnI, CK-MB, and LDH. Dapagliflozin also reduced serum levels of IL-1β, reduced expression of myocardial inflammation-related proteins, and inhibited cardiac caspase-1 activity. The treatment restored autophagy flux and promoted the degradation of autophagosomes. Relief of inflammation relied on autophagosome phagocytosis of NLRP3 and autophagosome clearance after lysosome improvement. 10 μM dapagliflozin reduced intracellular Ca^2+^ and Na^+^ in primary cardiomyocytes, and increasing NHE1 and NCX expression mitigated dapagliflozin effects on autophagy.

**Conclusion:** Dapagliflozin protects against myocardial ischemia/reperfusion injury independently of its hypoglycemic effect. High-dose dapagliflozin pretreatment might limit NLRP3 inflammasome activation and mediate its selective autophagy. Dapagliflozin directly acts on cardiomyocytes through NHE1/NCX.

## Introduction

Ischemic heart disease (IHD) is the primary cause of morbidity and mortality worldwide (1), imposing tremendous pressures on the healthcare system and economic development (2). It is estimated that by 2030, IHD will be the second leading cause of death worldwide (3). The primary treatment for IHD is coronary reperfusion (percutaneous coronary intervention, thrombolysis, and coronary bypass surgery) to restore the blood supply to the ischemic myocardium (4). However, sudden reperfusion may cause additional damage (5), commonly known as myocardial ischemia/reperfusion injury (I/R). Prevention of the injury after myocardial reperfusion is a challenge.

Dapagliflozin (DAPA), a sodium-glucose co-transporter-2 (SGLT-2) inhibitor, is a new type of antidiabetic medication that reduces blood glucose by inhibiting SGLT-2 in the proximal tubules of the kidney (6). Unlike other hypoglycemic drugs, dapagliflozin rarely causes hypoglycemia, even in non-diabetic patients (7). Studies suggested that SGLT-2 inhibitors are associated with significantly improved cardiovascular outcomes in patients with type 2 diabetes (T2DM) (8), including improving myocardial structure (9) and cardiac function (10), inhibition of cardiac inflammation (10), reduction of oxidative stress (11) and myocardial cell apoptosis (10), protection of mitochondrial function (9) and maintaining ion balance in isolated cardiomyocytes (12). For these reasons, in patients without T2DM who suffer from cardiovascular diseases like heart failure, SGLT-2 inhibitors might benefit (13, 14). We hypothesized that SGLT-2 inhibitors would meliorate myocardial ischemia/reperfusion injury.

Activation of inflammation and release of inflammatory cytokines aggravate myocardial I/R injury (15). Inflammasomes are an essential component of the inflammatory response, leading to the production of pro-inflammatory cytokines, such as NLRP3-related inflammasomes, which are composed of NLRP3 (NACHT, LRR, and PYD domains containing protein three), procaspase-1, and ASC (apoptosis-associated speck-like protein containing a CARD). Inflammasomes may activate caspase-1 to cleave IL-1β (16). Autophagy is a self-protection mechanism in eukaryotes under normal conditions (17). In autophagy, cytoplasmic components are transferred to lysosomes for degradation. Autophagy has been reported in myocardial I/R injury (18). In addition, autophagy might eliminate inflammatory stimuli and degrade NLRP3-related inflammasomes to retard inflammation (19). The inflammasomes are ubiquitinated, recruited to the autophagic adapter protein P62, and then transported to autophagosomes to complete the degradation process (20). It is worth noting that autophagy and inflammation are double-edged swords for cardiac I/R: they can decrease or increase cardiac I/R depending on their degree of activation. This study aimed to determine whether dapagliflozin would protect against myocardial I/R injury and reduce cardiac inflammation stimuli and if this result could be achieved by modulating autophagy.

## Materials and Methods

### Animals

All related operations involving animals follow the “Guidelines for Care and Use of Laboratory Animals” (NIH Publication No.85-23, revised 1996) published by the US National Institutes of Health (Bethesda, MD). And approved by the Animal Care and Use Committee at the Wenzhou Medical University (wydw2020-0634). All Male C57/BL6 mice (6–7 weeks old and 20–25 g weight) were purchased from the SLAC Laboratory Animal Center of Shanghai (Shanghai, China).

### Myocardial Ischemia/Reperfusion Model

The myocardial I/R model was operated as our previous study (21). In detail, the mice were first anesthetized with isoflurane and intubated. Then a thoracotomy was performed to expose the heart in the third or fourth rib. The left anterior descending coronary artery was ligated with a 7-0 silk thread to cause myocardial ischemia. After 30 min of ischemia, the silk thread was untied and the blood was reperfused for 4 h. Mice in the sham group underwent the same operation but were not ligated. The operation uses an animal anesthesia machine/ventilator from RWD Life Science, the pressure is adjusted to 0.4 after isoflurane perfusion, flow rate two, stroke volum (μl) 300, strokes/min 180. The isoflurane pumping was stopped immediately after the start of reperfusion, and the air was maintained until awakening.

### High-Throughput Sequencing

We selected a total of 6 mouse heart samples, of which three were treated by sham operation, and three were ischemia for 30 min and reperfused for 4 h. After the samples were collected, they were transported to Beijing on dry ice in accordance with the biological sample storage requirements, and Beijing Novogene completed high-throughput second-generation sequencing.

### Animal Grouping and Pretreatment

After all C57/BL6 mice were reared to 7 weeks, they were randomly divided into five groups: (a) Sham group-mice were subjected to sham operation and treated with vehicle (saline); (b) I/R group- mice were subjected to I/R intervention and treated with vehicle (saline); (c) I/R+CQ group-mice were subjected to I/R intervention after intraperitoneal injection of chloroquine and treated with vehicle (saline); (d) I/R+DAPA group-mice were subjected to I/R intervention and treated with dapagliflozin (40 mg/kg/day); (e) I/R+ DAPA+CQ group-mice were subjected to I/R intervention after intraperitoneal injection of chloroquine and treated with dapagliflozin (40 mg/kg/day). Treatment with dapagliflozin (MCE, China, 40 mg/kg/day) or vehicle by gavage was started 7 days before the cardiac I/R intervention. The dose of chloroquine (Sigma, USA) is 10 mg/kg, which is injected intraperitoneally 10 min before each operation, which has been mentioned in our previous article (21).

### Detection of Myocardial Infarction Size and Risk Area

Evans Blue/Triphenyltetrazolium chloride (TTC, Sigma, USA) staining was used to detect the myocardial infarct size induced by I/R injury. The coronary artery was ligated again, and the abdominal aorta was injected with 200 μL of 2% Evans Blue, the heart was immediately taken out, placed in a mold for sectioning, and then incubated in 1% TTC staining solution at 37°C for 30 min, after finishing overnight in 4% paraformaldehyde and filming. The images were analyzed using Image Pro software (Media Cybernetics, Inc. Bethesda, MD), and infarct size was calculated using a method as previously described (21), infarct size was compared when area at risk that were similar among groups.

### Serum Biochemical Analysis

Blood was taken 4 h after reperfusion, and the serum was separated by centrifugation at 7,500 rpm for 15 min, and immediately detected or temporarily frozen at −80°C. Serum TnI concentration was measured using an enzyme-linked immunosorbent assay (ELISA) measurement of Troponin I kit (Elabscience, E-EL-M0086c). Serum LDH activity was measured using a Lactate dehydrogenase (LDH) assay kit (Nanjing jiancheng, China). Serum creatine kinase-MB (CK-MB) was measured with a creatine kinase MB isoenzyme Assay Kit (Nanjing jiancheng, China).

### Detection of Secreted IL-1β

Serum concentrations and cell culture supernatant medium level of IL-1β were measured by the corresponding ELISA kits (Elabscience, E-EL-M0037) according to the manufacturer's instructions.

### Detection of Caspase-1 Activities

The cardiac activity of caspase-1 was calculated via Colorimetric Assay Kit (Solarbio, China). First, the total protein was extracted according to the instructions and formulated into a 2 mg/ml system.

### Myocardial Tissue and Cellular Immunofluorescence

Immunofluorescence staining is used in myocardial tissues and cells. Use frozen tissue sections or 4% paraformaldehyde-fixed cells, rupture the cell membrane with 0.3% Triton for 10 min, block with 5% BSA for 1 h at room temperature, and then incubate with the corresponding primary antibody at 4°C overnight. Incubate with the corresponding secondary antibody for 1 h in the dark the next day, and finally add DAPI to stain the nucleus. After completion, add anti-fluorescence quenching solution and take pictures under the microscope (Leica, Germany).

### Cardiomyocyte Primary Culture (PCCMs)

Isolate primary cardiomyocytes from the heart of C57/BL6 mice born within 1–3 days. The specific method can refer to the study by Ehler et al. (22). The detailed steps for isolation of primary cardiomyocytes are written in the Supplementary Material.

### Hypoxia/Re-Oxygenation (H/R) Model

These PCCMs were then used to simulate the H/R model (22). Hypoxic conditions was performed in a hypoxic incubator with an N_2_ concentration of 94% and a O_2_ concentration of 1% and a CO_2_ concentration of 5% for 6 h in medium deprived of serum. Then PCCMs were replaced with normal medium and put into ordinary incubators (CO_2_ concentration of 5% and O_2_ concentration of 21%) for 18 h to simulate reoxygenation injury. For *in vitro* experiments, DAPA(10 μM) was administered for 1 h (23) and CQ was administered for 24 h at 10 μM (22) in advance. Before starting H/R, replace with a new medium.

### Cell Viability and Toxicity Detection

The PCCMs were plated in 24-well plates and treated separately. After processing, use the Cell Counting Kit-8 (Beyotime, China) to measure the number of viable cells according to the manufacturer's instructions. At the same time, the upper medium of PCCMs after treatment was tested for cytotoxicity with LDH Assay Kit.

### Hoechst 33342/PI Staining Assay

Hoechst 33342/PI (Beyotime, China) has been reported to be used to label dead or apoptotic cells. We seeded PCCMs on cell slides in 24-well plates. After completing the corresponding treatments, we added Hoechst 33342 and PI working solution in order according to the manufacturer's instructions. Finally, we added the anti-fluorescent quenching solution and took it under a fluorescent microscope for observation.

### Lysotracker Red Stain

Lysotracker Red can be selectively retained in the acidic lysosomes, thereby achieving specific fluorescent labeling of lysosomes (24). In our experiments, we used Lysotracker Red (Beyotime, China) to detect the number of intact lysosomes in an H/R model simulated by PCCMs.

### Co-immunoprecipitation

PCCMs lysates were centrifuged for 10 min at 14,000 g at 4°C. 500 μL of the lysate (1000 μg of total protein) was added to the monoclonal primary antibody of the target protein at 4°C overnight. The next day, agarose beads were added and incubated for 3 h. The agarose beads were washed 3 times and then mixed with SDS for western blot analysis. The above reagents were all purchased from absin (abs955, China).

### Fluo-4AM and SBFI AM Measurement

First, the PCCMs are grouped and processed as before. After the H/R treatment, dilute Fluo-4AM (Beyotime, China) and SBFI AM to working concentrations according to the instructions, add them to the culture medium, incubate at 37°C for 30 min, wash with PBS and then put them back into the cell incubator for 30 min, and select the 334/505 wavelength for detection.

### Transfection Protocol

For overexpression plasmid transfection, 1 μg plasmid was added in 500 μl of Opti-MEM, followed by addition of 3 μl of Lipofectamine RNAiMAX in 500 μl of Opti-Mem and incubated for 5 min. The two mixtures were pooled and incubated further for 10 min at RT. The respective transfection mixture was then added to the cells and mixed by gently swirling the plate. The plate was then incubated at 37°C for 18 h in a 5% CO2 incubator. After incubation, media was added to the cells, and the cells were further incubated until harvest i.e., 48 h from the beginning of transfection.

### Western Blot Analysis (WB)

Myocardial tissue/cell total protein preparation and concentration determination kits are from Beyotime (Shanghai, China). Proteins of different molecular weights were separated on SDS-PAGE gels, transferred to 0.22 μm PVDF membranes, and blocked with skimmed milk powder then incubated with primary antibodies of NLRP3 (A12694, ABclonal), Caspase-1 (22915-1-AP, proteintech), ASC (abs135824, absin), IL-1β (16806-1-AP, proteintech), Cleaved-IL-1β (A1112, ABclonal), Atg-5 (12994T, CST), Beclin-1 (3738S, CST), P62 (23214S, CST), LC3B (ab243506, abcam), LAMP1 (3243S, CST), LAMP2 (66301-1-Ig, proteintech),CTSD (21327-1-AP, proteintech), CTSB (12216-1-AP, proteintech), and GAPDH (10494-1-AP, proteintech) at 4°C overnight. After incubating with the corresponding secondary antibody the next day, protein bands were scanned by a ChemiDoc MP device (Bio-Rad, Hercules, CA, USA). Finally, ImageJ software (NIH, USA) was used for analysis.

### Statistical Analysis

Data were presented as mean ± SD, and were analyzed using SPSS version 21.0 (IBM, Armonk, NY, USA). All the data we used were normally distributed. A two-tailed unpaired Student's *t*-test was conducted to compare two experimental groups or one way analysis of variance (ANOVA) followed by Duncan's T3 multiple-range test was used for the comparison of more than two groups. *P* < 0.05 were considered as statistically significant.

## Results

### Dapagliflozin Treatment Attenuates Myocardial Ischemia/Reperfusion Injury *in vivo* and *in vitro*

**Figure 1:**
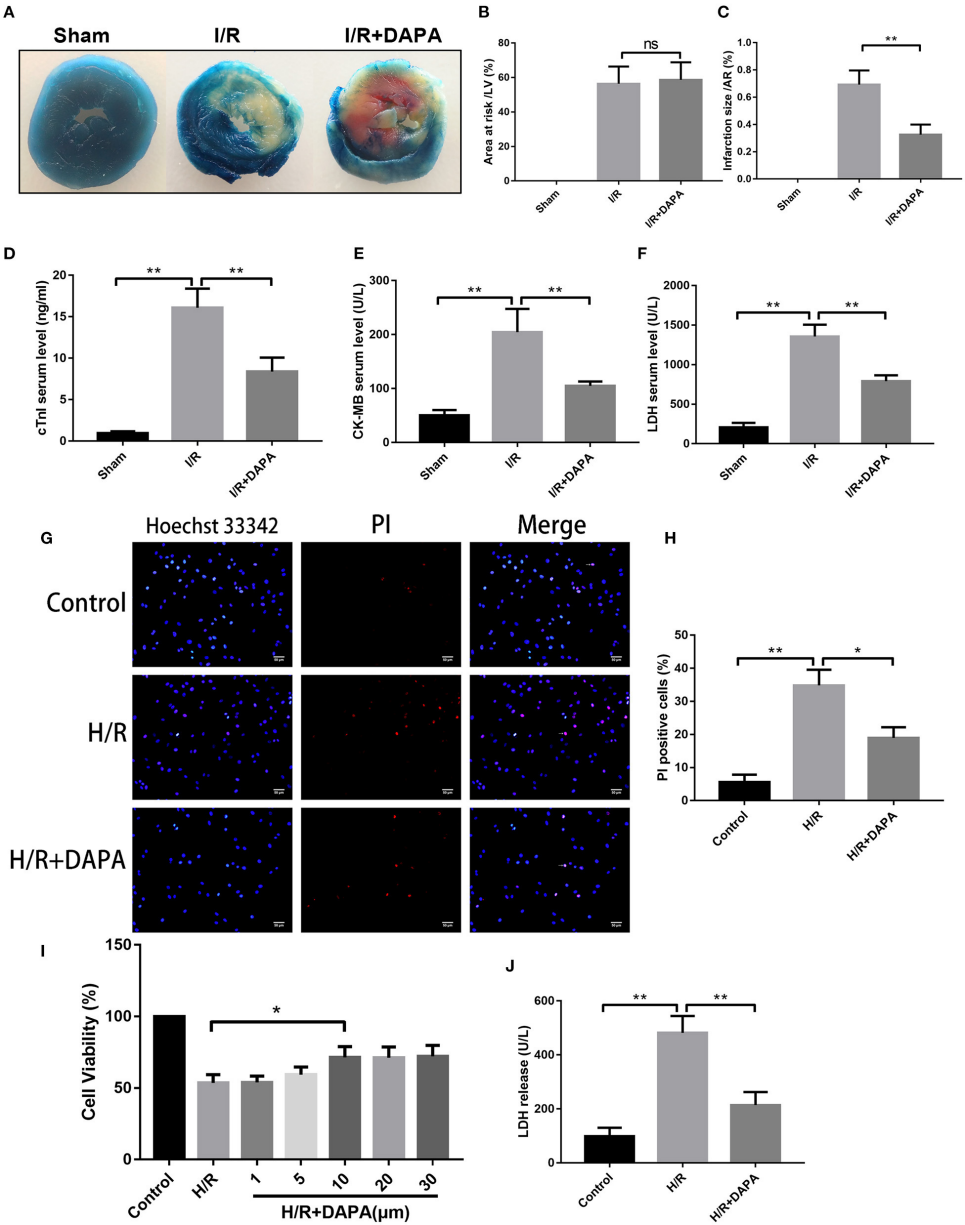
Dapagliflozin treatment attenuates myocardial ischemia/reperfusion injury *in vivo* and *in vitr*o. **(A)** 6-7w mice were administered DAPA or vehicle control, established I/R model, and measured the infarct size of each group using TTC. **(B,C)** Comparison of the myocardial infarction area in each group, *n* = 3. **(D–F)** cTnI, CK-MB, and LDH levels in mouse serum, *n* = 9. **(G)** Red indicates PI staining and blue indicates Hoechst staining. **(H)** PI staining positive ratio, *n* = 3. **(I,J)** Lactate dehydrogenase (LDH) and cell viability assay, *n* = 9. Data are means ± SDs, *P < 0.05; **P < 0.01.

We first evaluated the effect of DAPA on myocardial I/R injury by assessing myocardial infarct size. After DAPA treatment, the area of myocardial infarction in mice was significantly decreased for area at risk that were similar among groups (Figures 1A–C). DAPA significantly decreased cTnI, CK-MB, and LDH levels after I/R (Figures 1D–F).

Coincidentally, we also found that DAPA treatment significantly improved cell viability of PCCMs after hypoxia/re-oxygenation injury (H/R). The optimal concentration of DAPA was 10 μM (Figure 1I). Subsequent treatment with 10 μM DAPA reduced LDH in the cell supernatant (Figure 1J) and a significant decrease in myocardial cell death (Figures 1G,H).

### Dapagliflozin Treatment Attenuates the Inflammatory Response in Myocardial I/R Injury

**Figure 2:**
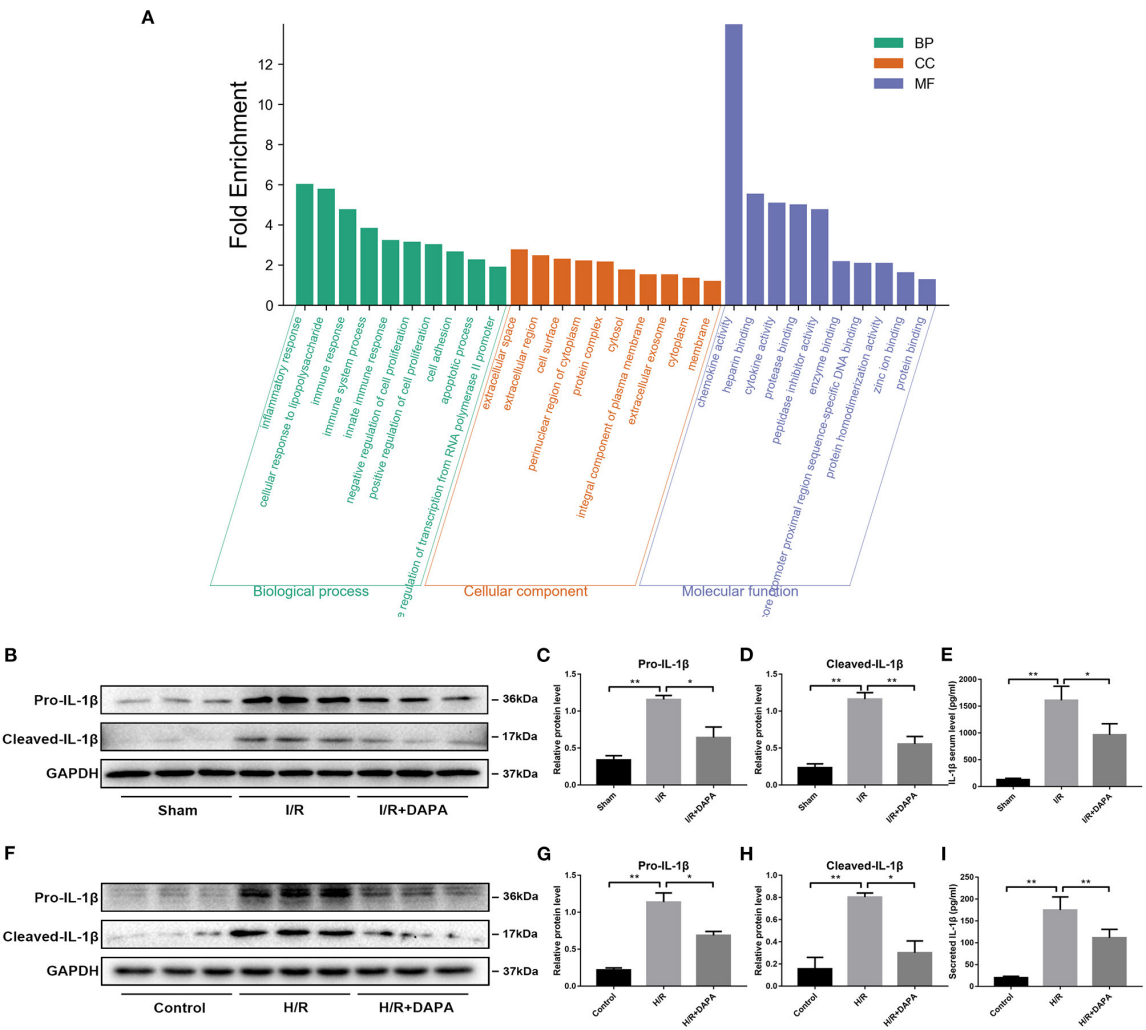
Dapagliflozin treatment attenuates the inflammatory response in myocardial I/R injury. **(A)** GO function enrichment after I/R myocardial high-throughput sequencing, fold change ≥1.5 and adjusted *p* < 0.05, *n* = 3. **(B)** Western blot detection was performed from LV protein extracts from each group *in vivo*. **(C,D)** Western blot analysis of Pro-IL-1β and Cleaved-IL-1β *in vivo, n* = 3. **(E)** Serum IL-1β levels, *n* = 9. **(F)** Western blot detection was performed from PCCMs protein extracts from each group *in vitro*. **(G,H)** Western blot analysis of Pro-IL-1β and Cleaved-IL-1β *in vitro, n* = 3. **(I)** Secretion of IL-1β from cell supernatants, *n* = 9. Data are means ± SDs, *P < 0.05; **P < 0.01.

Many factors cause myocardial I/R injury; to explore which factor is the main factor in our model, we performed high-throughput sequencing on the I/R injury area. Sequencing results suggest that inflammatory response is the central part of the biological process (Figure 2A).

We speculated that DAPA treatment would reduce the inflammatory response in I/R injury. First, we detected inflammatory proteins in the tissues and found that DAPA treatment significantly reduced the expression levels of Pro-IL-1β and Cleaved-IL-1β (Figures 2B–D). We also detected the secretion level of IL-1β in the serum of mice. It was found that DAPA treatment also reduced the secretion of IL-1β in the serum of I/R mice (Figure 2E). Coincidentally, DAPA also improved H/R injury of PCCMs *in vitro*. DAPA treatment decreased expression of Pro-IL-1β and Cleaved-IL-1β *in vitro* (Figures 2F–H) and reduced the secretion of IL-1β in the PCCMs culture medium (Figure 2I).

### Dapagliflozin Can Inhibit the Assembly and Activation of NLRP3 Inflammasome

**Figure 3:**
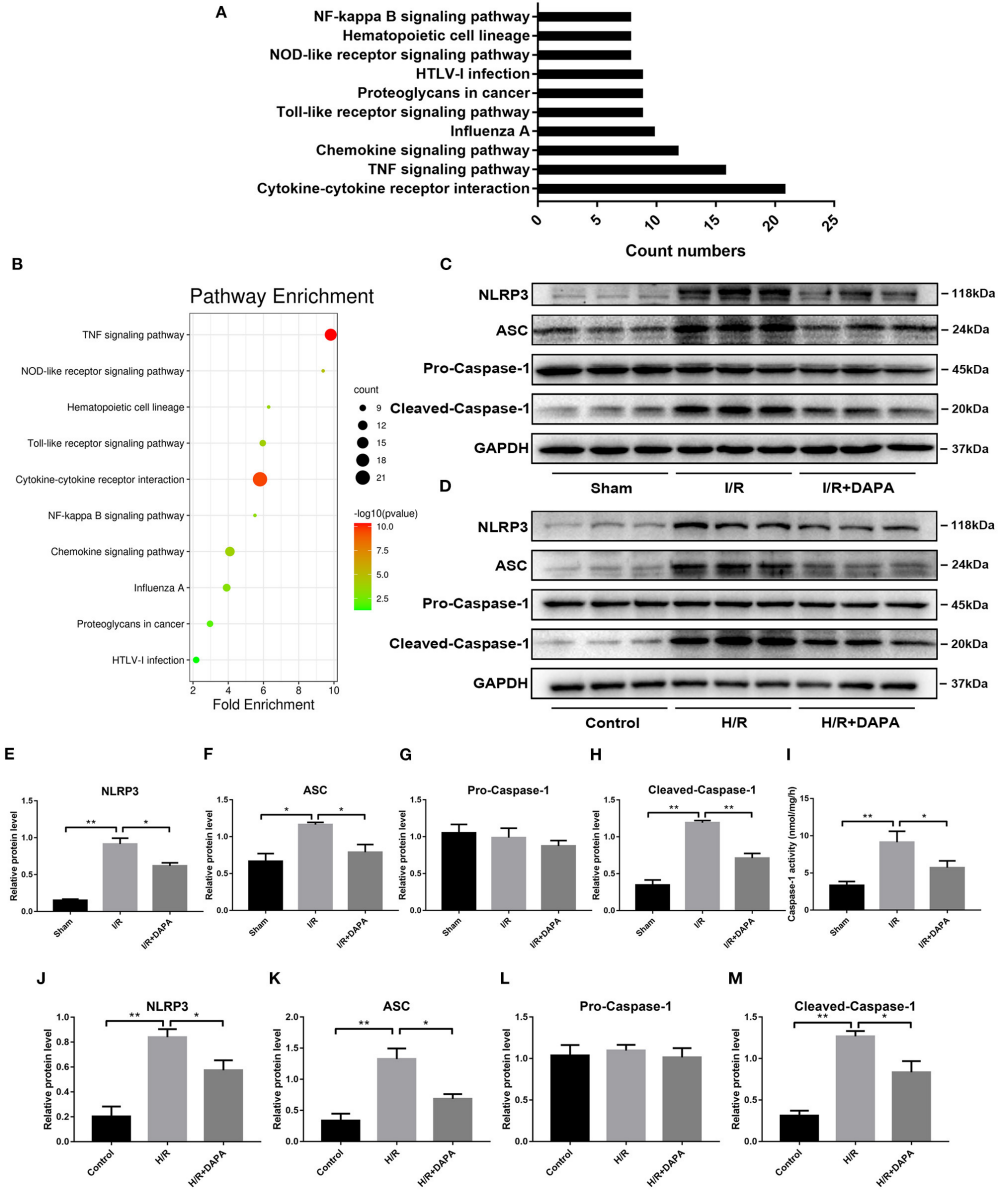
Dapagliflozin can inhibit the assembly and activation of NLRP3 inflammasome. **(A,B)** KEGG pathway enrichment after I/R myocardial high-throughput sequencing, fold change≥1.5 and adjusted *p* < 0.05, *n* = 3. **(C)** Western blot detection was performed from LV protein extracts from each group *in vivo*. **(D)** Western blot detection was performed from PCCMs protein extracts from each group *in vitro*. **(E–H)** Western blot analysis of NLRP3, ASC, Pro-Caspase-1, Cleaved-Caspase-1 *in vivo, n* = 3. **(I)** Caspase-1 activity in myocardium tissues, *n* = 9. **(J–M)** Western blot analysis of NLRP3, ASC, Pro-Caspase-1, Cleaved-Caspase-1 *in vitro, n* = 3. Data are means ± SDs, *P < 0.05; **P < 0.01.

Through sequencing, we have found that the inflammatory response dominates in our I/R model, and DAPA treatment inhibits this inflammatory response. In order to explore how inflammation is activated here, we performed KEGG pathway analysis on the sequencing results and screened the top 10 using “count number” as the standard and found that the signaling pathways directly related to inflammation are “NF-kappa B” and “NOD-like receptor” (Figure 3A). And through screening with “fold enrichment” as the standard, it is found that “NOD-like receptor” is more important (Figure 3B).

So we selected NLRP3 inflammasome as the verification target. By detecting the level of NLRP3 inflammasome-related protein, we found that after DAPA treatment, the expression of NLRP3 and ASC were reduced (Figures 3C–F,J,K), and the production of Cleaved-caspase-1 was also significantly inhibited (Figures 3C,D,G,H,L,M). On the other hand, the enzyme activity of caspase-1 activated during I/R injury also decreased significantly after DAPA treatment (Figure 3I).

### Dapagliflozin Treatment Restores Autophagy Flux Following Ischemia/Reperfusion Injury *in vivo* and *in vitro*

**Figure 4:**
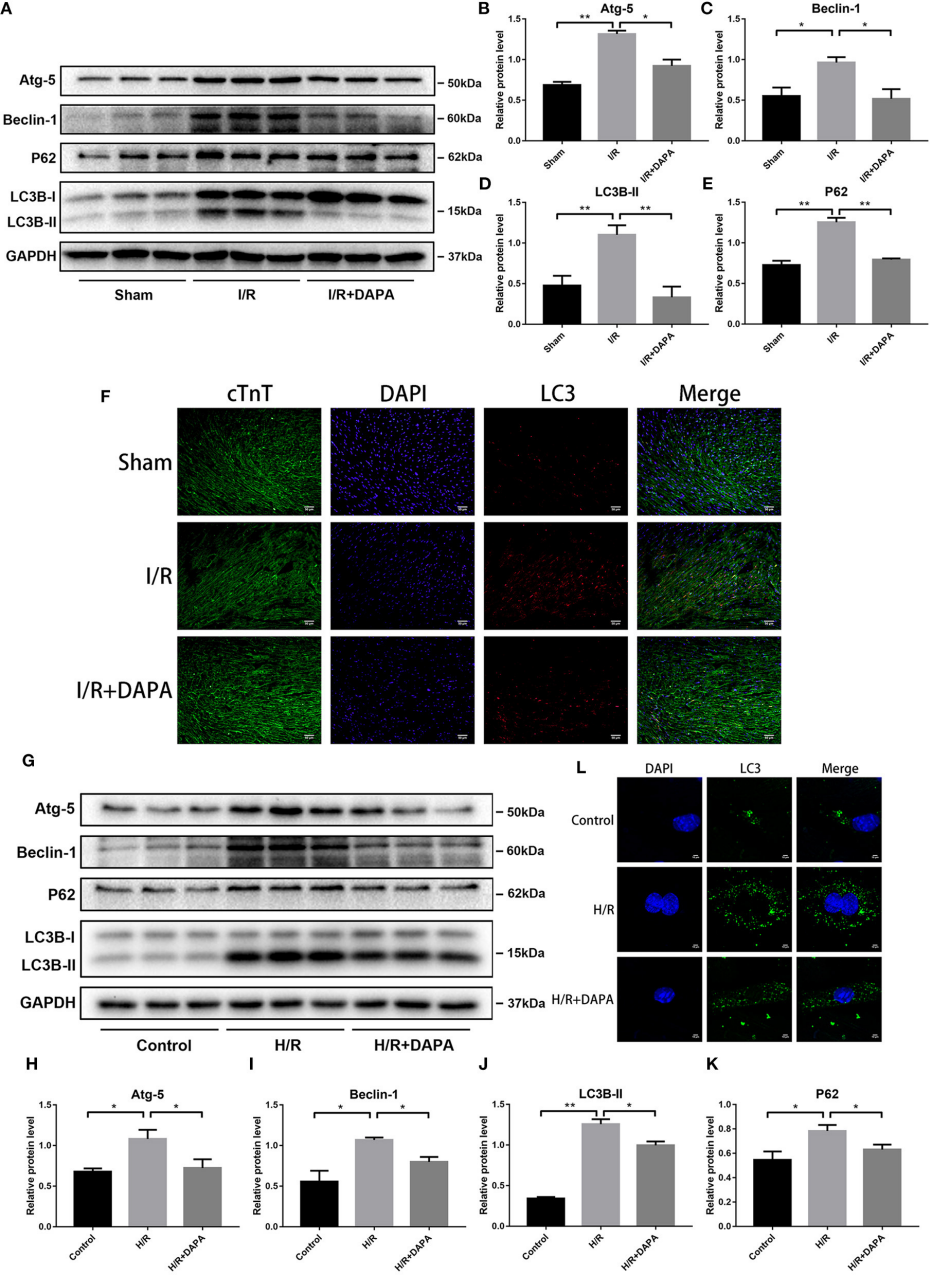
Dapagliflozin treatment restores autophagy flux following ischemia/reperfusion injury *in vivo and in vitro*. **(A)** Western blot detection was performed from LV protein extracts from each group *in vivo*. **(B–E)** Western blot analysis of Atg-5, Beclin-1, LC3B-II and P62 *in vivo, n* = 3. **(F)** LC3 immunofluorescence staining to detect autophagosome formation *in vivo, n* = 3. **(G)** Western blot detection was performed from PCCMs protein extracts from each group *in vitro*. **(H–K)** Western blot analysis of Atg-5, Beclin-1, LC3B-II and P62 *in vitro, n* = 3. **(L)** LC3 immunofluorescence staining to detect autophagosome formation in PCCMs, *n* = 3. DAPA was pretreated for 1 h at 10 μM *in vitro*. Data are means ± SDs, *P < 0.05; **P < 0.01.

Recently, autophagy has been implicated in I/R (25) and may be related to inflammation (19). We analyzed the expression of autophagy-related proteins by Western blot analysis to determine the effect of DAPA on autophagy in myocardial I/R injury. The results showed that LC3B-II, a representative protein of autophagy, was significantly increased in I/R, accompanied by an increase of Atg-5 and Beclin-1, which are related to autophagosome production (26). P62, a ubiquitinated protein that may indicate autophagic flux (26), was also increased (Figures 4A–E,G–K). The formation of autophagosomes was increased, and the destruction of autophagosomes was reduced, which led to the accumulation of autophagosomes.

DAPA treatment reduced the expression of LC3B-II, Atg-5, beclin-1, and P62 (Figures 4A–E,G–K). We next explored whether the reduction of autophagosomes after DAPA treatment was due to inhibition of autophagosome production, increased destruction, or both. To show that this phenomenon occurs in myocardial cells, we co-stained LC3 and cTnT (cardiac-specific markers) and measured their relative concentrations by immunofluorescence (Figure 4F). The results were consistent with those of Western blot assays. When PCCMs were stained for LC3 and imaged by immunofluorescence microscopy, the results were the same: pre-treatment of DAPA reduced the accumulation of autophagosomes (Figure 4L).

Moreover, the expression of P62 was reduced after the addition of DAPA. P62 is a selective autophagy receptor and is degraded by autophagy; thus, increased levels of p62 reflect the inhibition of autophagy (27). Combined with our previous results, we speculated whether there is a relationship between this blocked autophagy flux and increased inflammation and whether hindering autophagosome degradation affects the inflammation change in I/R injury.

### Dapagliflozin Mediated Cardiomyocyte Protection Depends on the Expected Clearance of Autophagosomes *in vivo*

One of the normal elements of autophagy flux, the successful degradation of autophagosomes, requires the participation of normally-functioning lysosomes (28). We used chloroquine (CQ) to disrupt lysosomal function and block autophagosome degradation (22).

**Figure 5:**
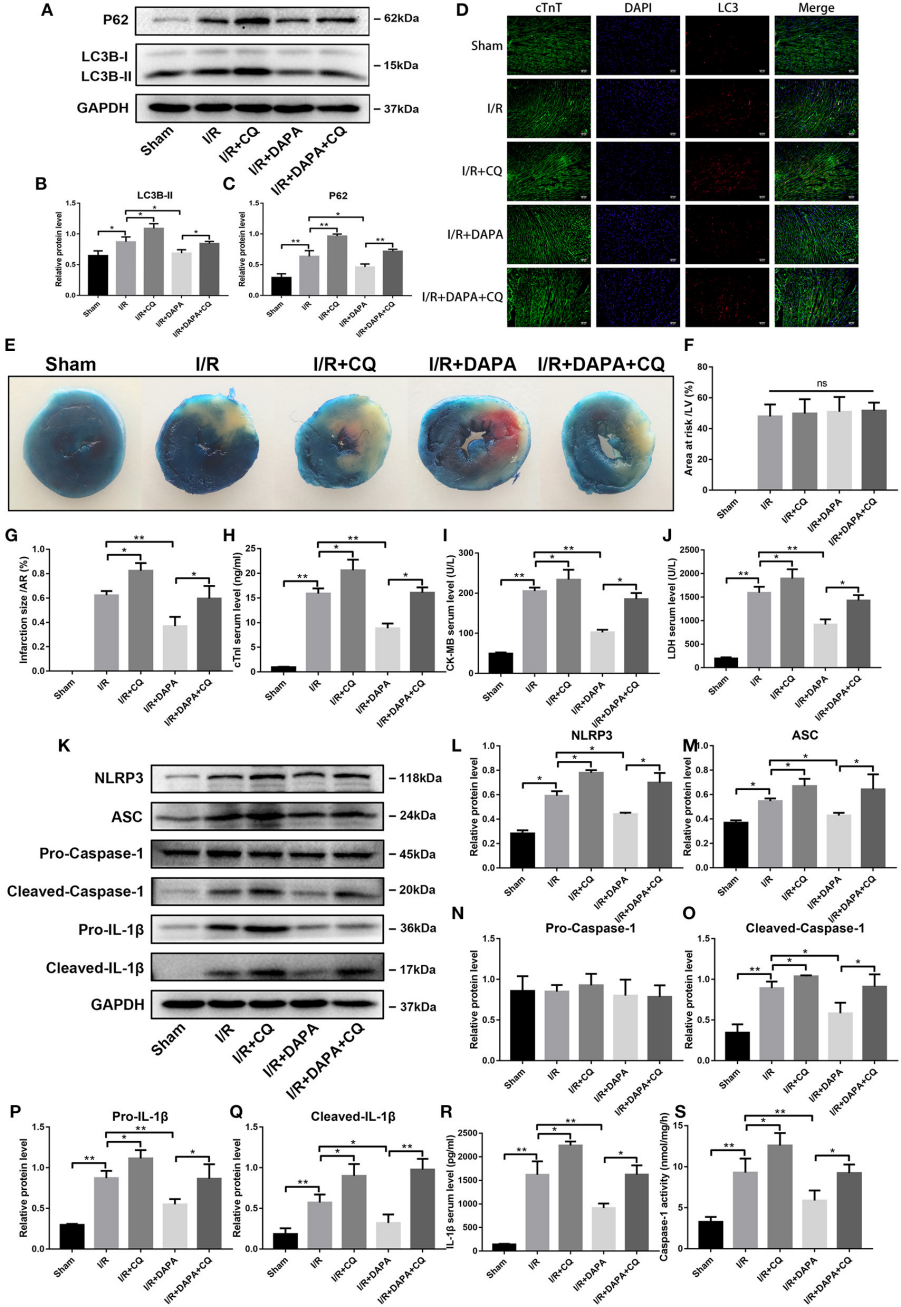
Dapagliflozin mediated cardiomyocyte protection depends on normal clearance of autophagosomes *in vivo*. **(A)** Western blot detection was performed from LV protein extracts from each group *in vivo*. **(B,C)** Western blot analysis of LC3B-II and P62 *in vivo, n* = 3. **(D)** LC3 immunofluorescence staining to detect autophagosome formation *in vivo, n* = 3.**(E)** C57/bl6 mice were administered DAPA, CQ or vehicle control, established I/R model, and measured the infarct size of each group using TTC. **(F,G)** Comparison of the myocardial infarction area in each group, *n* = 3. **(H–J)** cTnI, CK-MB, and LDH levels in mouse serum, *n* = 9. **(K)** Western blot detection was performed from LV protein extracts from each group *in vivo*. **(L–Q)** Western blot analysis of NLRP3, ASC, Pro-Caspase-1, Cleaved-Caspase-1, Pro-IL-1β and Cleaved-IL-1β *in vivo, n* = 3. **(R)** Serum IL-1β levels, *n* = 9. **(S)** Caspase-1 activity in myocardium tissues, *n* = 9. Data are means ± SDs, *P < 0.05; **P < 0.01.

After CQ exposure, we first detected autophagy-related proteins by Western blotting to determine whether CQ inhibition was achieved. Compared with the DAPA group, the DAPA + CQ group showed a significant increase in LC3B-II (Figures 5A,B), indicating an increase in autophagosome accumulation. Similarly, in the DAPA + CQ group, levels of P62 were significantly higher than those in the DAPA group (Figures 5A,C). Therefore, we inferred that the degradation of autophagosomes was reduced after the addition of CQ. We concluded that the use of CQ in our model successfully inhibited the degradation of autophagosomes. We also examined whether this phenomenon occurred in myocardial cells. Using tissue immunofluorescence technology, we found that the results on both sides were consistent (Figure 5D). We also discovered that in the comparison between the I/R group and the I/R + CQ group, the expression of LC3B-II and P62 in the I/R + CQ group was further enhanced. Blocked degradation of autophagosomes when I/R occurred appeared to be incomplete.

We then asked whether I/R damage changed after using CQ. We observed that damage in the DAPA + CQ group increased following the addition of CQ. Compared with the DAPA group, the DAPA + CQ group increased the myocardial infarction area (Figures 5E–G). Similarly, we tested myocardial infarction markers in the serum, including cTnI, CK-MB, and LDH, and found that the treatment effect of the DAPA group was reversed by CQ (Figures 5H–J). Compared to the I/R group, the I/R + CQ group results were exacerbated to varying degrees (Figures 5E–J). Therefore, the elimination of autophagosomes plays a more critical role in myocardial I/R injury. Impaired clearance will increase I/R damage. Conversely, promoting the elimination of autophagosomes can reduce I/R injury, and DAPA may play an important role here.

What is the relationship between clearance of autophagosomes and I/R inflammation? When autophagosome clearance was blocked by CQ, inflammation in the DAPA + CQ group was significantly greater than that in the DAPA group. The most direct manifestation was the increased expression of pro-IL-1β and cleaved-IL-1β (Figures 5K,P,Q). Serum IL-1β secretion increased after CQ administration (Figure 5R). The expression of NLRP3 and ASC also changed similarly as inflammation (Figures 5K,L,M). With cleaved-caspase-1 (Figures 5K,N,O,S), which represents the protein that inflammasomes activate for further function, inflammation in the I/R + CQ group was further exacerbated than the I/R group. The observations suggest that clearance of autophagosomes in I/R injury is also closely related to the occurrence and progression of inflammation.

Combining these observations, we found that DAPA may play a role by restoring autophagy flux and autophagosome degradation, which was damaged by I/R injury.

### Dapagliflozin Mediated Cardiomyocyte Protection Relies on Normal Clearance of Autophagosomes in PCCMs

**Figure 6:**
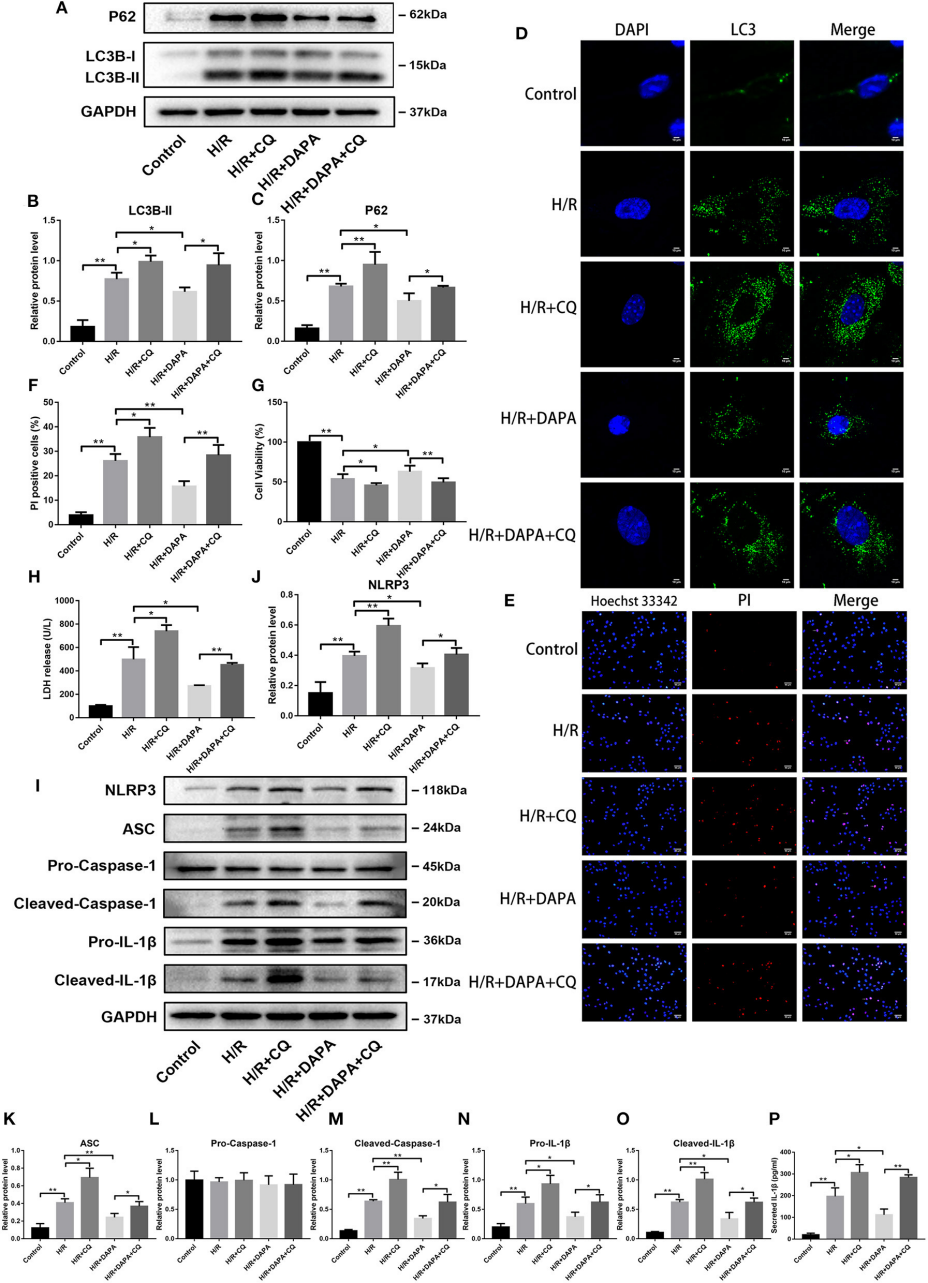
Dapagliflozin mediated cardiomyocyte protection relies on normal clearance of autophagosomes in PCCMs. **(A)** Western blot detection was performed from PCCMs protein extracts from each group *in vitro*. **(B,C)** Western blot analysis of LC3B-II and P62 *in vitro, n* = 3. **(D)** LC3 immunofluorescence staining to detect autophagosome formation in PCCMs, *n* = 3. **(E)** Red indicates PI staining and blue indicates Hoechst staining. **(F)** PI staining positive ratio, *n* = 3. **(G,H)** Lactate dehydrogenase (LDH) and cell viability assay, *n* = 9. **(I)** Western blot detection was performed from PCCMs protein extracts from each group *in vitro*. **(J–O)** Western blot analysis of NLRP3, ASC, Pro-Caspase-1, Cleaved-Caspase-1, Pro-IL-1β and Cleaved-IL-1β *in vitro, n* = 3. **(P)** Secretion of IL-1β from cell supernatants, *n* = 9. DAPA was pretreated for 1 h at 10 μM. Data are means ± SDs, *P < 0.05; **P < 0.01.

Next, we explored whether these results would occur in isolated myocardial cells using CQ and monitoring marker molecules related to autophagy. As shown in Figures 6A–D, when the pathway of autophagosome degradation was inhibited, the number of autophagosomes in PCCMs increased. Coincidentally, under H/R conditions, autophagosome clearance was blocked incompletely, which helps to clarify whether the changes *in vivo* and *in vitro* were consistent.

We tested whether damage after H/R injury changed after CQ inhibited autophagosome clearance in PCCMs. The addition of CQ further aggravated the damage of H/R, which can be seen from the comparison between the H/R and H/R + CQ groups (Figures 6G,H). Conversely, when comparing the DAPA + CQ group with the DAPA group, CQ eliminated the improvement attributable to DAPA (Figures 6G,H). Hoechst/PI double-stained PCCMs also showed the same change in the percentage of PCCMs deaths (Figures 6E,F). To further explore whether H/R injury and DAPA-related changes are related to inflammation, we examined the expression of inflammation-related proteins. As expected, CQ reversed the DAPA induced reduction of inflammation and aggravated H/R-induced inflammation, manifested by upregulation of pro-IL-1β and cleaved-IL-1β protein levels (Figures 6I,N,O) and increased IL-1β secretion in the cell supernatant (Figure 6P). In addition, observing the NLRP3 inflammasome index, the results showed that after CQ suppressed autophagosome clearance, levels of NLRP3, ASC, and cleaved-caspase-1 increased (Figures 6I–M).

These observations suggest that H/R-impaired autophagosome clearance cause damage, which DAPA can partially offset by promoting autophagosome clearance.

### Dapagliflozin Mediated Cardiomyocyte Protection Is Related to Selective Degradation of NLRP3 by Autophagosomes in PCCMs

How does DAPA reduce I/R-induced inflammation and improve I/R injury by promoting autophagy? Autophagy can remove toxic waste, including some compounds that affect excessive inflammation (29). In addition, inflammasome components, including NLRP3, can be cleared by autophagy (19). Based on these reports, we believe that autophagosomes in our model system clear NLRP3 and that DAPA may play an important role.

**Figure 7:**
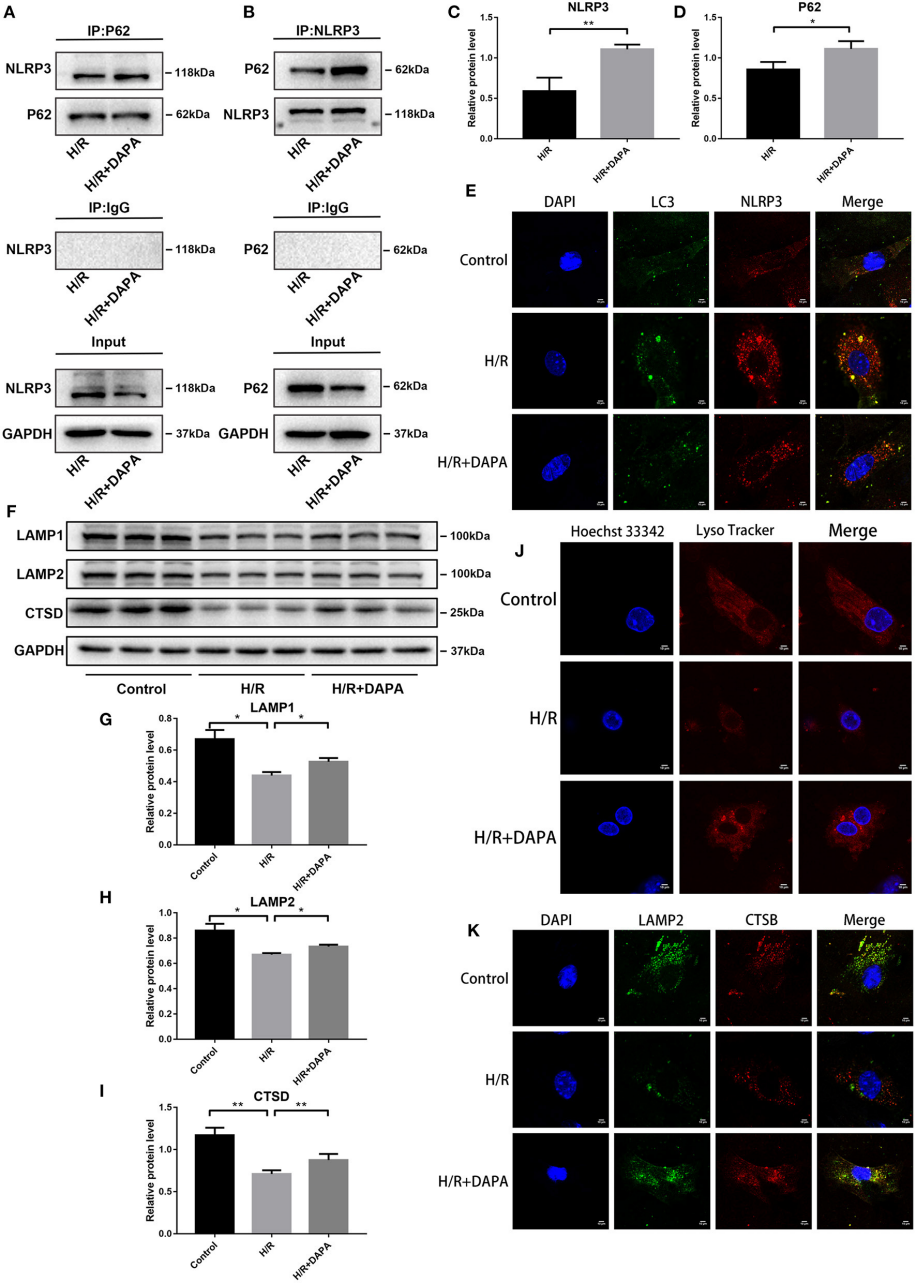
Dapagliflozin improves lysosomal function and accelerates the selective degradation of NLRP3 in PCCMs. **(A,B)** Combination of NLRP3 protein with P62 was identified by Co-IP. **(C,D)** Western blot analysis of Co-IP, *n* = 3. **(E)** Fluorescence co-localization of LC3 and NLRP3, *n* = 3. **(F)** Western blot detection was performed from PCCMs protein extracts from each group *in vitro*. **(G–I)** Western blot analysis of LAMP1, LAMP2 and CTSD, *n* = 6. **(J)** Lyso Tracker Red staining detected lysosomes in PCCMs, *n* = 3. **(K)** Fluorescence co-localization of LAMP2 and CTSB, *n* = 3. DAPA was pretreated for 1 h at 10 μM. Data are means ± SDs, *P < 0.05; **P < 0.01.

We used co-immunoprecipitation and fluorescence co-localization to investigate this phenomenon further. Through Western blot analysis, we observed an interaction between P62 and NLRP3, possibly promoted by DAPA, in the H/R and H/R + DAPA groups (Figures 7A–D). Fluorescence results identified co-localization of autophagosome marker LC3 and inflammasome marker NLRP3 (Figure 7E). These findings suggest that eliminating autophagosomes may remove the inflammasome component NLRP3, and the application of DAPA may accelerate the phagocytosis of NLRP3 by autophagosomes.

### Dapagliflozin Accelerates Autophagosome Clearance by Reducing Lysosomal Destruction in PCCMs

Autophagosomes phagocytose the inflammasome component NLRP3, and although DAPA may strengthen this process, it is insufficient to degrade the autophagosomes adequately. We have shown that DAPA can promote the clearance of autophagosomes in I/R, but the successful clearance of autophagosomes requires normal lysosomes for proper degradation. Disruption of lysosomal function or impaired lysosomal degradation capacity will affect autophagy flux (28).

To determine if DAPA improved the function of lysosomes in I/R, we used Lysotracker red probe for acid staining to mark normal lysosomes. As shown in Figure 7J, the number of lysosomes with an acidic environment decreased in the H/R group but increased in the H/R + DAPA group. In addition, we detected some indicators of lysosomes at the protein level, such as LAMP1, which closely tracks lysosome numbers, and LAMP2, a critical protein of autophagosome-lysosome fusion (18), that showed reduced expression in the H/R group but were upregulated in the H/R + DAPA group (Figures 7F–H). CTSD (Cathepsin D) and CTSB (Cathepsin B), typical lysosomal labeled proteases, are involved in the degradation of autophagosomes (30). We found that CTSD expression was downregulated in the H/R group but increased after DAPA treatment (Figures 7F,I). We used LAMP2 to co-stain with CTSB and observed a decrease in fluorescence in H/R; the overlap between LAMP2 and CTSB was also reduced (Figure 7K). These results suggest that (a) the lysosomal membrane permeability changed in H/R, resulting in a decrease in the number of lysosomes with a typical acidic environment, and (b) some cathepsins in the lysosome were released into the cytoplasm. Although these factors may be responsible for reducing autophagosome degradation, DAPA appears to attenuate this damage.

### Dapagliflozin Protects Cardiomyocytes by Inhibiting NHE1 and NCX

**Figure 8:**
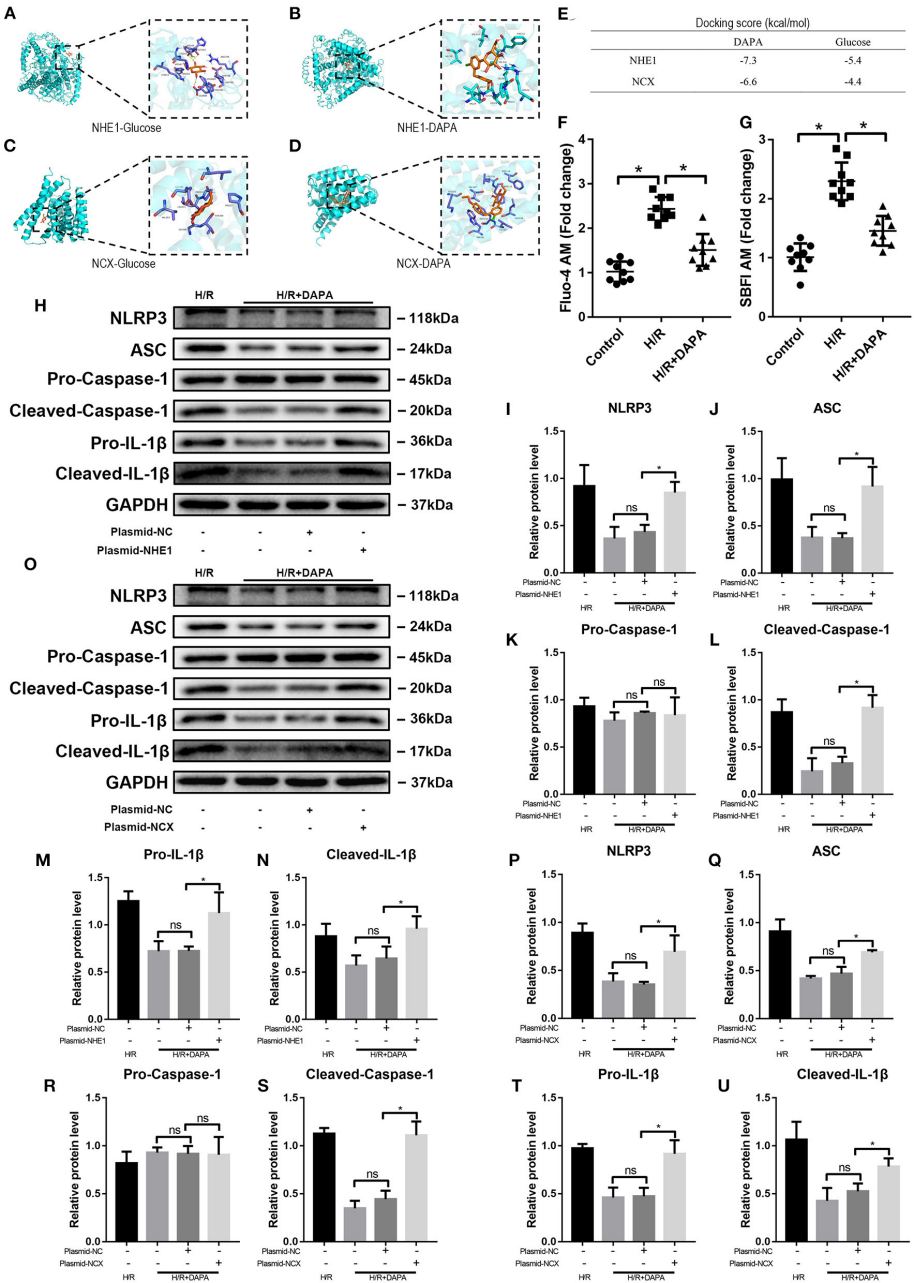
Dapagliflozin protects cardiomyocytes by inhibiting NHE1 and NCX in PCCMs. **(A–D)** Molecular docking with Auto Dock Tools. **(E)** Docking score. **(F)** Ca^2+^ concentration change in PCCMs, *n* = 9. **(G)** Na^+^ concentration change in PCCMs, *n* = 9. **(H–N)** Western blot detection was performed from PCCMs protein extracts from each group with overexpression plasmid-NHE1 *in vitro, n* = 3. **(O–U)** Western blot detection was performed from PCCMs protein extracts from each group with overexpression plasmid-NCX *in vitro, n* = 3. DAPA was pretreated for 1 h at 10 μM. Data are means ± SDs, *P < 0.05; *P* < 0.01. ns: not statistically.

Studies have suggested that SGLT-2i can act on NHE1 (sodium hydrogen ion transporter 1) on cardiomyocytes and inhibit its activity (12), thereby producing physiological effects; others believe that it is myocardial protection produced by NCX (sodium-calcium ion transporter) effect (31). We used Auto Dock Tools to perform molecular simulations on dapagliflozin and NHE1 (PDBID: 3v5s) and NCX (I-TASSER) and found that dapagliflozin and NHE1, and NCX have binding ability (Figures 8A–E). Further, we observed that after DAPA treatment, Ca^2+^ and Na^+^ in PCCMs have been effectively alleviated.

We used plasmids to upregulate the activities of NHE1 Figures 8F,G and NCX in PCCMs to prove whether DAPA's intervention is related to these two receptors. The results showed that as the activity of NHE1 increased during H/R injury, the assembly and activation of NLRP3 inflammasomes also increased (Figures 8H–N), indicating that the increased activity of NHE1 eliminated the treatment brought by DAPA. Similarly, the upregulation of NCX activity also showed the above results (Figures 8O–U).

## Discussion

**Figure 9:**
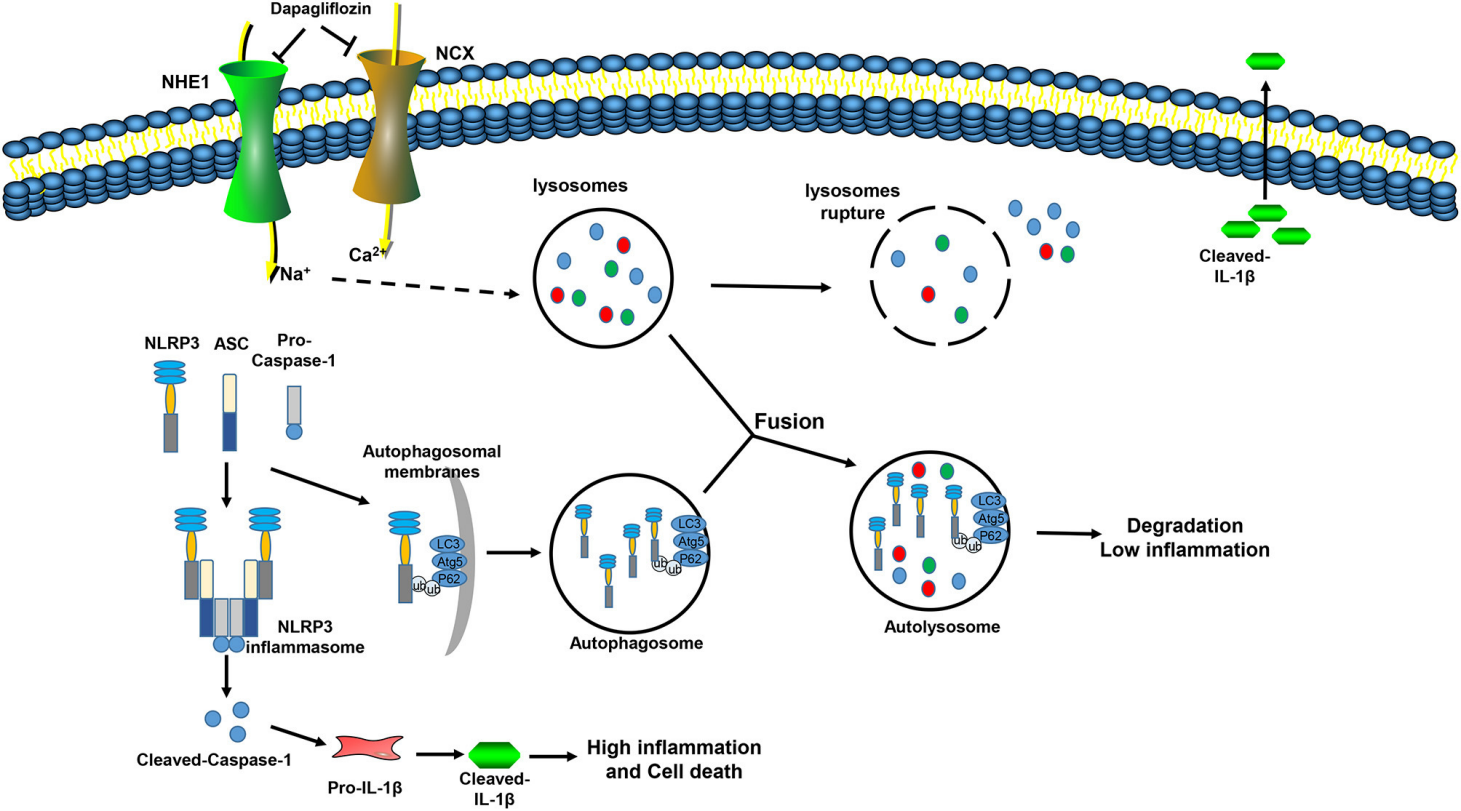
Mechanism model of dapagliflozin reducing myocardial I/R injury. During I/R injury, Na^+^ and Ca^2+^ overload in myocardial cells leads to increases in the permeability of the lysosomal membrane, cathepsin in the lysosome leaks out, autophagosome clearance are inhibited, and the NLRP3 inflammasome is assembled and activated, which aggravates the inflammation and promotes cell death. Dapagliflozin inhibits the activity of NHE1 and NCX in the myocardium, relieves Na+, Ca2+ overload, improves lysosome function, promotes autophagosome degradation, promotes the interaction of P62 and NLRP3, inhibits inflammasome assembly, inhibits inflammation activation, reduces inflammatory damage, and reduces cell death.

Our current research provides new insights into the role of DAPA in protecting against myocardial I/R injury in mice. We found: (a) Impaired autophagosome clearance during myocardial I/R in mice, which increases the inflammatory response and aggravates myocardial damage; (b) DAPA attenuates the enlargement of infarct size by restoring autophagy flux and inhibiting inflammation; (c) DAPA provides cardioprotection by promoting the degradation of the inflammasome component NLRP3 during autophagy; (d) DAPA promotes autophagosome degradation by reducing lysosomal damage and protecting lysosomal function; and (e) DAPA acts directly on cardiomyocyte NHE1/NCX (Figure 9).

Dapagliflozin, empagliflozin, and canagliflozin are SGLT-2 inhibitors. SGLT-2 is mainly expressed in the kidney but not in the heart (32). Numerous reports suggest that SGLT-2 inhibitors may inhibit inflammation, especially in patients with T2DM. In the early stages of diabetes, long-term use of empagliflozin can inhibit macrophage infiltration, reduce oxidative stress, and suppress inflammation (11). Studies suggested that empagliflozin can directly act on the myocardium through NHE1 while acting on the myocardium through blood sugar levels (33). Dapagliflozin and canagliflozin may alter macrophage polarization and inhibit the production of pro-inflammatory factors such as IL-1β, IL-6, and TNF-α (34). In addition, SGLT-2 inhibitors may reduce the production of NLRP3/ASC inflammasome, thereby suppressing inflammatory responses (10), independent of lowering blood glucose.

Recent studies indicate that SGLT-2 inhibitors may work by acting on autophagy. For example, empagliflozin can increase the LC3B II/I ratio and decrease the expression of P62 (35). However, some studies have noted that SGLT-2 inhibitors can upregulate autophagy-related ubiquitinated protein P62 (36). In our *in vivo* and *in vitro* studies, dapagliflozin downregulated P62 and reduced the expression of LC3B II. It is not yet known what caused this difference. It is not clear how SGLT-2 inhibitors regulate autophagy, but autophagy activation is often related to nutritional deprivation, involving AMPK (37) (adenosine monophosphate-activated protein kinase), SIRT1 (38) (sirtuin-1), HIF-1α (39), and HIF-2α (40) (hypoxia-inducible factors). Coincidentally, there have been many reports of interactions between SGLT-2 inhibitors and these proteins. For example, canagliflozin and empagliflozin increase SIRT1 expression (41), and changes in SIRT1 will affect levels of HIF-2α (42). Empagliflozin can activate AMPK and HIF-1α (43). These exciting experiments may suggest that there are more links between SGLT-2 inhibitors and autophagy. Some studies have noted that empagliflozin is inefficient at activating AMPK at a lower concentration (23), even if this concentration is available in some studies. We speculated that the activated AMPK protein had been degraded (44) at the time of our measurement due to the different injury times, which produced the opposite result.

The typical inflammatory response is mediated by activated caspase-1, and the assembly of NLRP3 inflammasome is the prerequisite for caspase-1 to be activated. Following the production of inactive IL-1β by pro-inflammatory stimulation, inactive IL-1β must undergo inflammasome treatment before it can mature and be secreted. Many stimuli can induce inflammation and activation of the NLRP3 inflammasome, including I/R (15), corroborating our findings. In our study, the inflammatory factors pro-IL-1β and cleaved-IL-1β have been changing along with inflammasome-related indicators. Autophagy may be used to control the elimination of unnecessary and excessive inflammation (20). One possible mechanism for reducing inflammation is the degradation of inflammasomes and related components through the action of P62. Our research also demonstrated the effect of autophagy on inflammation. When we inhibited the elimination of autophagosomes, inflammation increased, but promoting the degradation of autophagosomes reduced the inflammatory response. Furthermore, we showed that autophagosomes could degrade the inflammasome-constituent protein NLRP3. When this core component, which is used to assemble inflammasomes, is eliminated, there is less processing to promote the maturation of inflammatory cytokines.

Treatment with dapagliflozin significantly improved cell death, possibly in many forms. Studies have mentioned that dapagliflozin improves cardiomyocyte apoptosis and mitochondrial function (45). It has been reported that dapagliflozin affects energy metabolism, and then affects death outcome through autophagy flux (46), or improves inflammation-related cell apoptosis (10), inhibits STAT3 pathway, etc (34). Most of the researches involve the changes of SGLT-2i on cell energy metabolism, and it is speculated that it may be related to Na+/Ca2+. Although we are only involved in inflammation at present, inflammation can also induce cell death, such as pyroptosis (47). What's more, the NLRP3 inflammasome is also the main regulator of pyroptosis in I/R injury. On the other hand, most of the current researches are still focused on the chronic treatment of drugs. Although some studies mention that the acute treatment of SGLT-2i can also bring improvement (45, 48), it has been found that SGLT-2i needs to be used before ischemia occurs or in the middle of ischemia can save the death of myocardium. This undoubtedly increases the difficulty of using drugs for treatment. It may be a better choice that early preventive use for high-risk groups.

There are several limitations to our research. First, we cannot rule out the effects of other mechanisms that protect I/R damage in addition to the mechanisms examined here, such as the secretion level of insulin/glucagon. Second, although we focused on eliminating autophagosomes, whether the induction of autophagy can be further induced or inhibited will require further study. Furthermore, our results suggest that DAPA processing promotes the labeling of NLRP3 by P62, but it is not clear how this step is completed. Fourth, since cardiomyocytes do not express SGLT-2, we searched for NHE1 and NCX as new targets for dapagliflozin through molecular docking, but this requires more precise interaction to prove their connection, such as SPR (Surface Plasmon Resonance) technology and gene knockout and overexpression *in vivo*. Finally, treatment with 40 mg/kg/d DAPA was approximately 20x higher than the allometric-adapted dose used in human clinical trials. The 10 μM DAPA in our cellular studies is 20x higher than the maximal concentration of DAPA observed in human serum. Therefore, we cannot exclude that our results are primarily caused by these high dosages and are challenging to translate to human conditions. Further research is necessary to examine this finding in more depth. For example, biological materials should be used to transport dapagliflozin; therefore, the heart is treated with high doses of medications alone.

In summary, DAPA can improve I/R damage, reduce infarct size, and improve cardiac function in non-diabetic mice. The protective effect is accomplished, at least in part, by the selective autophagy degradation of the inflammasome component NLRP3, reducing the maturation and secretion of inflammatory factors. At the same time, although SGLT-2 does not exist in cardiomyocytes, dapagliflozin can directly act on NHE1/NCX in the myocardium.

## Data Availability Statement

The raw data supporting the conclusions of this article will be made available by the authors, without undue reservation.

## Ethics Statement

The animal study was reviewed and approved by Animal Care and Use Committee at the Wenzhou Medical University (wydw2020-0634).

## Author Contributions

Y-WY: designed the experiment, completed the research, analyzed the experimental data, and wrote the manuscript. J-QQ, K-YH, LQ, SL, Y-BW, F-NR, LW, and Y-YZ: participated in the experiments. Y-JX and K-TJ: participated in experimental design and manuscript revision. All authors have read and approved the final manuscript.

## Funding

This study was funded by the National Natural Science Foundation of China (no. 81573185), Research Fund for Lin He's Academician Workstation of New Medicine and Clinical Translation (no. 17331201, 18331207) and Chinese Society of Integrated traditional Chinese and Western Medicine and Scientific Research Fund of HeHuang Pharmaceutical Industry (no. 2019002) and the Scientific Research Foundation of the Science and Technology Department of Zhejiang Province (No. LQ21H020011).

## Conflict of Interest

The authors declare that the research was conducted in the absence of any commercial or financial relationships that could be construed as a potential conflict of interest.

## Publisher's Note

All claims expressed in this article are solely those of the authors and do not necessarily represent those of their affiliated organizations, or those of the publisher, the editors and the reviewers. Any product that may be evaluated in this article, or claim that may be made by its manufacturer, is not guaranteed or endorsed by the publisher.
