# Supplementary material for: Sodium-Glucose Co-transporter-2 Inhibitor of Dapagliflozin Attenuates Myocardial Ischemia/Reperfusion Injury by Limiting NLRP3 Inflammasome Activation and Modulating Autophagy
Source: Front Cardiovasc Med. 2022 Jan 10;8:768214. doi: 10.3389/fcvm.2021.768214 (PMC8785320; doi:10.3389/fcvm.2021.768214)
Supplement: Supplementary file 1 [file Table_1.DOCX]

**Supplementary materials**

Sodium-glucose cotransporter-2 inhibitor of dapagliflozin attenuates myocardial ischemia/reperfusion injury by limiting NLRP3 inflammasome activation and modulating autophagy

1. **Cardiomyocyte primary culture (PCCMs)**
   1. Take out the suckling mice within 1-3 days of birth, and after initial disinfection with 75% ethanol, cut open the chest with ophthalmological scissors, squeeze out the heart of the suckling rat, take out the heart with ophthalmic forceps, and immediately put it in the sterile Wash in PBS, wash on ice, and transfer to new PBS after washing.
   2. Replace with new ophthalmic forceps, remove the large blood vessels in the auricle, and wash the blood stains on the heart with PBS.
   3. Wash again, then transfer the heart into a 1.5ml EP tube, add a small amount of separation solution, and cut it with new ophthalmological scissors (0.5-1mm^3^).
   4. Add the chopped heart into a centrifuge tube containing 10ml of separation solution, about 10 hearts/tubes of suckling mice
   5. Shake overnight at 4°C for about 12 hours, depending on the wire drawing situation.
   6. Coat the petri dish with the prepared gelatin overnight one day in advance and place it in the incubator. When the separation is over the next day, take out the petri dish to remove the gelatin, and turn on the UV to dry in the operating table.
   7. Take out the heart tissue from the previous night. After the tissue mass settles naturally, aspirate the supernatant and leave about 1ml residual liquid. Add 5ml digestion solution and 5ml F-12K medium containing 20mM BDM, and pipette continuously for 1min. , 37℃ water bath for 2min (no overtime).
   8. The above-mentioned centrifuge tube is shaken and digested on a constant temperature shaker (speed ≤60) at 37°C for 20-30min (10 per tube is generally 20min).
   9. Put a 40-100μm sterile filter on a 50ml centrifuge tube, wet the filter with 2-3ml of F-12K medium containing 20mM BDM in advance, and then pipette the digested tissue block 10-20 times per tube. Until the tissue mass is evenly dispersed in the supernatant.
   10. Aliquot the digested cell suspension into two 15ml centrifuge tubes, centrifuge at 1000rpm for 5min, remove the supernatant, and resuspend the cells with 10ml seeding medium.
   11. The cells are planted in a common cell culture dish, transferred into a constant temperature cell incubator and cultured for 30 minutes for differential adherence.
   12. After the differential adhesion is completed, gently rinse the culture dish with the supernatant, and then transfer the culture solution to the culture dish that was coated with gelatin in advance and air-dried; the previous ordinary culture dish adhered to myocardial fibroblasts , You can add fibroblast culture medium to culture for 1-2 days, observe the content of cardiomyocytes and adjust the time of differential adhesion.
   13. The gelatin-coated petri dishes were cultured in an incubator for 12-18 hours to allow cardiomyocytes to adhere to the wall and develop their morphology.
   14. The next day, the cardiomyocytes were cultured with maintenance medium, and the maintenance medium was replaced every 24h.
   15. Follow-up processing and use.
2. Supplementary figure


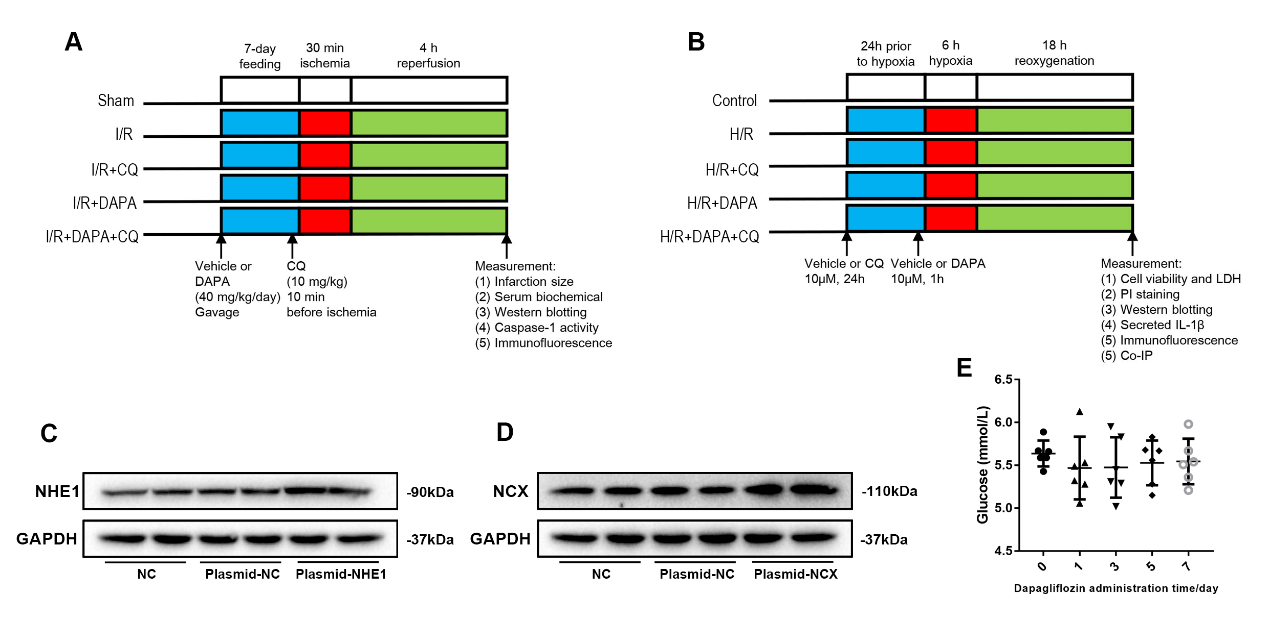


**Supplementary figure legend:**

(A) Diagram of in vivo experiment. Sham (20/20), I/R (21/30), I/R+CQ (19/30), I/R+DAPA (25/30), I/R+DAPA+CQ (22/30). (B) Diagram of in vitro experiments. (C) Western blot verification of NHE1 plasmid. (D) Western blot verification of NCX plasmid. (E) Blood glucose monitoring in mice. The blood glucose was measured before dapagliflozin feeding and 1, 3, 5, and 7 days after feeding. The measurement time was 10 am every day.
